# Acceptability and impact on health-related markers of a sustainable dietary pattern: results from a pilot randomized controlled cross-over study

**DOI:** 10.1007/s00394-026-04014-7

**Published:** 2026-06-19

**Authors:** Valentina Vinelli, Massimiliano Tucci, Daniela Martini, Marco Rendine, Samuele Venturi, Simone Perna, Giorgio Gargari, Giacomo Mantegazza, Claudio Gardana, Alberto Battezzati, Alessandro Leone, Sabrina Sucato, Silvia Fustinoni, Simone Guglielmetti, Marisa Porrini, Cristian Del Bo’, Patrizia Riso

**Affiliations:** 1 Division of Human Nutrition, Department of Food, Environmental and Nutritional Sciences (DeFENS), Università degli Studi di Milano, Milano, Italy; 2 Division of Food Microbiology and Bioprocesses, Department of Food, Environmental and Nutritional Sciences (DeFENS), Università degli Studi di Milano, Milano, Italy; 3 Department of Biotechnology and Biosciences (BtBs), University of Milan Biococca, Milan, Italy; 4 Department of Food, Environmental and Nutritional Sciences (DeFENS), International Center for the Assessment of Nutritional Status and the Development of Dietary Intervention Strategies (ICANS-DIS), Università degli Studi di Milano, Milano, Italy; 5 Department of Clinical Sciences and Community Health, University of Milan, 20122 Milan, Italy; 6 IRCCS CA’ Granda Foundation, Policlinico Maggiore Hospital, Milan, Italy

**Keywords:** Sustainability, Planetary health diet, Plant-based dietary pattern, Nutrition, Acceptability

## Abstract

**Purpose:**

Well-balanced plant-based diets can promote health and reduce environmental impact. However, evidence from interventional studies remains limited. This pilot intervention study aimed to provide exploratory insights into potential challenges associated with the EAT-IT dietary pattern, an adaptation of the EAT-Lancet Healthy Reference Diet.

**Methods:**

Nine subjects (mean age 26 ± 2 years, 5 females) participated in a 6-week randomized controlled cross-over trial. Participants followed two isocaloric interventions: the EAT-IT dietary pattern and a control diet based on the Italian Food-Based Dietary Guidelines. Dietary intake was recorded using 7-day food records. Anthropometric measurements and metabolic parameters were collected according to standardized and validated protocols. Gut microbiota was analyzed through 16 S rRNA gene sequencing and taxonomic profiling. Acceptability was evaluated via a validated questionnaire.

**Results:**

Nutritional analysis showed that the EAT-IT pattern significantly increased fiber intake from 11.3 ± 5.2 to 15.1 ± 4.2 g/1000 kcal and ω-6 fatty acid intake from 5.7 ± 2.2 to 6.6 ± 1.9 g/day (*p* < 0.05 for interaction). Regarding metabolic markers, a significant within-group reduction (*p* < 0.05) was observed for fasting insulin (8.4 ± 2.2 to 6.5 ± 2.2 µU/mL) and HOMA1-IR (2.0 ± 0.6 to 1.5 ± 0.5). Changes in gut microbiota were also observed, specifically an increase in Bacteroides and a decrease in Coriobacteriaceae. While generally well-accepted, participants reported a higher perceived effort for EAT-IT, particularly regarding legume preparation.

**Conclusion:**

Despite the small sample size, this pilot study offers relevant insights into key aspects of sustainable plant-based diets, underscoring the necessity for further investigation.

## Introduction

Across Europe and globally, current dietary habits reveal prevalent consumption of low-quality diets high in energy, red meat, sugars, and salt, while on the other hand, the intake of nutrient rich foods like whole-grain cereals, fruits, vegetables, legumes, nuts, and milk remains for most individuals inadequate, falling below dietary recommendations [1, 2]. The Global Burden of Disease Study 2017 estimates that 22% of total deaths (11 million total) and 15% of disability-adjusted life-years (255 million total) in the adult population older than 25 years across 195 different countries are linked to dietary habits [3]. Consistent with previous evidence, the main dietary risk factors include excessive sodium intake and low consumption of whole grains, fruits, nuts, and vegetables [3].

Overall, it is widely acknowledged that dietary patterns rich in plant-based foods are favorably associated with several health markers associated with glucose-insulin homeostasis, blood lipids and lipoproteins, blood pressure, endothelial function, inflammation, and oxidative stress [4]. Additionally, these diets positively influence the composition and functionality of the gut microbiota, benefiting overall human health [5]. These effects are largely attributed to their high content of dietary fiber, micronutrients, and bioactive compounds such as polyphenols, carotenoids, and glucosinolates [6–11].

Plant-based foods also tend to have a lower environmental impact compared to animal source foods [12]. Switching to nutritionally balanced plant-based diets, reducing animal-source foods, could significantly decrease diet-related land use, greenhouse gas emissions, acidification, eutrophication and water use [12, 13]. Although these effects depend on several methodological aspects, including dietary composition and impact assessment parameters, current evidence suggests that the potential benefits for food system sustainability are substantial [14–17]. However, nowadays only a few of the current food-based dietary guidelines include instructions around environmental impacts of diet, which, if properly integrated, could accelerate the transition to a more sustainable food system [18]. This reinforces the need for the development and validation of dietary patterns promoting both human and planetary health, as also emphasized by multiple national and international organizations [18–21].

In response to these challenges, in 2019 the EAT-Lancet commission proposed a Healthy Reference Diet (ELHRD), also known as Planetary health diet, to be promoted at global level and developed based on a comprehensive framework that includes considerations on global population health promotion, food systems organization, and planetary boundaries [22]. This pattern consists primarily of whole grains, fruit, vegetables, legumes, nuts, unsaturated oils, low amounts of seafood, poultry, and little to no red meat, processed meat, added sugar, refined grains, or starchy vegetables [22]. However, despite the theoretical advantages on human and planet health, the significant substitution of animal source foods with plant-based foods raises important questions regarding the feasibility and acceptability of the diet and its actual impact on nutritional status in relation to specific micronutrients and bioavailability (e.g., calcium, iron, zinc, vit. B12) [23]. Because several nutrients are more concentrated and bioavailable in animal-source foods, such dietary shifts warrant careful evaluation to avoid unintended nutritional consequences [24, 25].

The ELHRD is envisioned as a reference dietary pattern to be adapted to align with the culinary traditions of various countries and different population nutritional requirements [22]. In line with this, we proposed a theoretical adapted version of the ELHRD, called EAT-IT, tailored to the Italian food context, and compared it in term of nutritional adequacy and environmental impact with a dietary pattern in line with the Italian Food-Based Dietary Guidelines (Italian FBDGs) [26]. These assessments revealed similarities for most nutrients, but also observed some differences, including higher energy from lipids and plant protein, higher fiber content, and lower levels of calcium in the EAT-IT dietary pattern compared to the Italian FBDGs [26]. From an environmental perspective, while the EAT-IT dietary pattern presented a lower carbon footprint compared to the pattern based on Italian FBDGs, it did not show the same advantage in terms of water footprint [27].

To further explore the development of sustainable dietary patterns that could be actually promoted at individual and household levels, in vivo studies are needed. Of particular relevance are the assessments of advantages, criticalities, and challenges in a real-life setting, while also evaluating acceptability aspects, which are essential for achieving sustainable diets according to the FAO’s definition [28]. Indeed, acceptability plays a crucial role in the feasibility of a sustainable healthy diet, especially when considering the differences between the dietary patterns intended to be promoted and habitual diets [29]. In addition to nutritional adequacy, food safety aspects, including potential exposure to contaminants, should also be considered.

To the best of our knowledge, human intervention studies aimed at investigating the different effects of dietary patterns based on the ELHRD on human health-related parameters are still lacking.

Within this context, a 6-week pilot randomized, controlled, crossover trial was designed as an exploratory step to assess acceptability of the EAT-IT dietary pattern and to study its effect on overall food and nutrient intake, metabolic and functional markers including inflammation and oxidative stress, as well as microbial ecosystem (i.e., gut microbiota composition) in a healthy population. Identifying these challenges in this homogeneous group will help refine the dietary pattern and of future large-scale interventions targeting more heterogeneous populations.

## Materials and methods

### Subject’s enrollment

For this study, 10 subjects were enrolled from the students and employees of the University of Milan through advertisements by using notice boards, emails, phone calls, and grapevine according to the inclusion and exclusion criteria. Consistent with the study rationale, the inclusion criteria required participants to be healthy and at least 18 years old. Exclusion criteria included: following specific diets (e.g., vegetarian or vegan diet); using medications or supplementation that could interfere with the study’s analysis; conditions necessitating specific diets or treatments (e.g., celiac disease) or conditions that could be adversely affected by dietary treatments (e.g., irritable bowel syndrome). Subjects’ eligibility was confirmed through a general anamnesis and interview/questionnaires about health status and lifestyle. Any uncertainties about a participant’s eligibility were resolved through consultation with the medical staff involved in the research. All participants signed the informed consent form before being enrolled. The protocol was approved by the Ethics Committee of the University of Milan (ref: 68/22 parere RISO_21.07.22).

### Experimental design

**Fig. 1:**
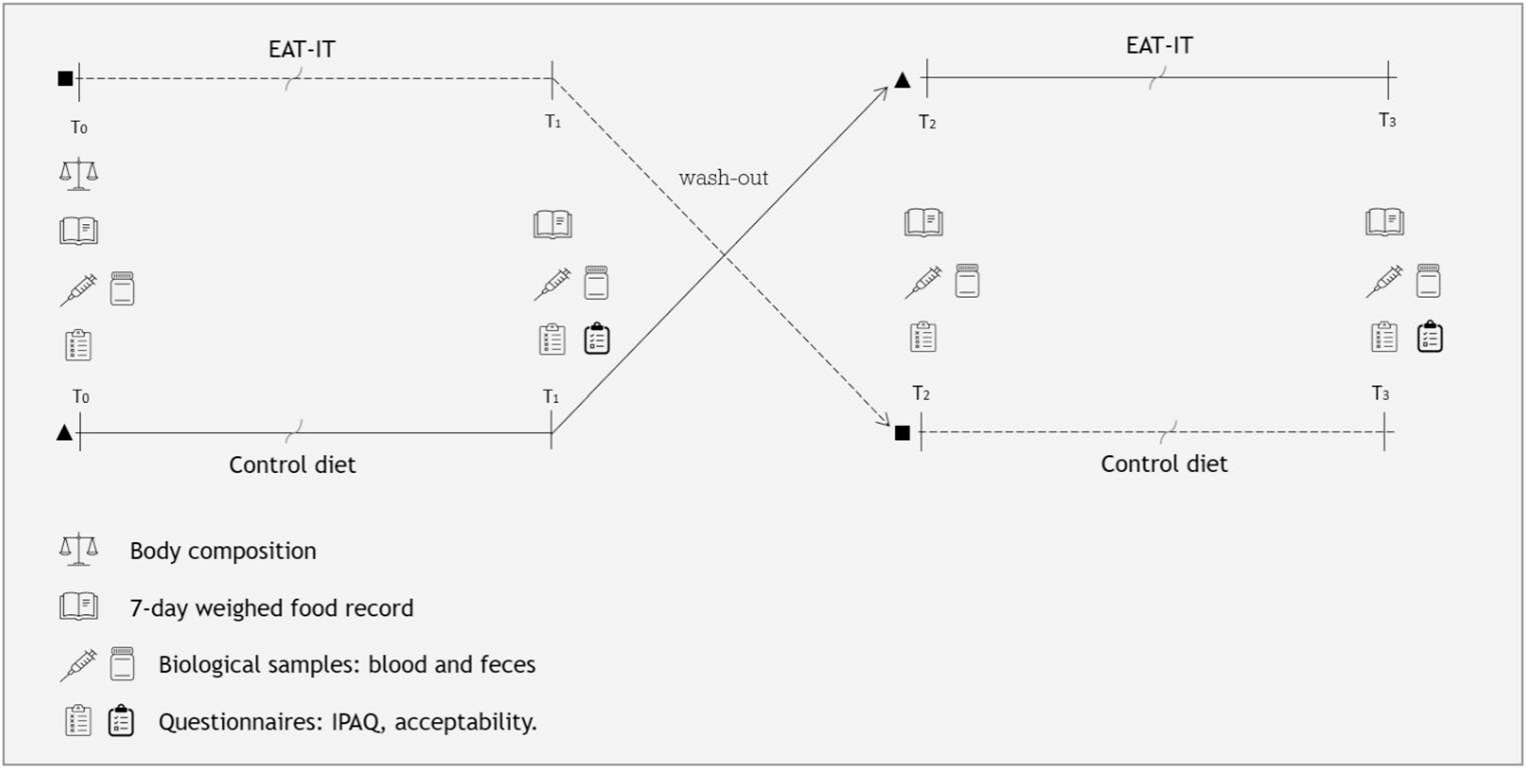
Study design: biological samples, food records and questionnaires collection. IPAQ, International physical activity questionnaire

This study was a pilot, single-blind, randomized controlled trial, with a crossover design, conducted between June and December 2022 at the Division of Human Nutrition of the Department of Food Environmental and Nutritional Sciences (DeFENS), University of Milan. According to the crossover design, subjects enrolled were expected to undergo both interventions. Thus, subjects were randomly divided (using a computer random number generator) into 2 sequences of intervention, separated by a washout period: EAT-IT/wash-out/Control, or Control/wash-out/EAT-IT, with a 1:1 ratio to overcome potential impact of period effects. Each intervention lasted 6 weeks, with a washout period of at least 6 weeks to account for carryover effects. Subjects enrolled were requested to fulfill a 7-day food record and provide biological samples (i.e., blood, urine and feces) at the beginning and at the end of each phase of intervention. A body composition assessment was also requested for the initial baseline characterization. Finally, different questionnaires related to lifestyle and dietary habits were collected at the beginning and at the end of each intervention. A representation of the study protocol is shown in Fig. 1.

### Dietary intervention

The EAT-IT dietary pattern represents a theoretically healthy and sustainable diet, developed based on the ELHRD. Details about the characteristics of this pattern were already reported elsewhere [26]. Briefly, the EAT-IT dietary pattern intervention was structured considering three main meals (i.e., breakfast, lunch and dinner) and 1–2 snacks of nuts. Breakfast included a portion of milk or derivatives, a portion of whole grains (i.e., wholemeal bread, whole meal rusks, oats, or corn flakes) and a portion of jam or fruit juice. Lunch and dinner included a portion of whole grains (i.e., whole meal rice or pasta, spelled, or wholemeal bread) or potatoes, a portion of animal or vegetable protein source food (i.e., beef, pork, or lamb; poultry, eggs, fish, or legumes), vegetables, extra virgin olive oil, and fruit, according to appropriate portion sizes and frequency of consumption based on the previous development of the pattern.

Dietary recommendations were provided to participants in the form of a digital scheme reporting portions sizes and frequencies of consumption for the different food categories, according to the different meals. Dietary schemes were personalized based on energy requirements and personal preferences (e.g., subjects do not drink milk). Details about the personalization process of the EAT-IT pattern are described in paragraph 2.4.

To increase adherence to the EAT-IT dietary pattern, during the study, different food products (e.g., canned legumes, wholegrain bread and pasta, 100% legume pasta, nuts) have been provided to participants during the study.

Control diet was constituted by a different scheme with portion sizes and frequencies of consumption based on Italian FBDGs [20], which already included tables reporting reference portion sizes and frequencies of consumption for 3 different energy targets (i.e., 1500, 2000, and 2500 kcal/day). A healthy control diet was used to ensure that differences did not simply reflect the contrast with suboptimal habitual diets but the specific characteristics of the EAT-IT dietary pattern. The main differences between the EAT-IT dietary pattern and the Italian FBDGs are a decreased consumption of some animal source foods (eggs, milk and derivatives, poultry, seafood), reduced amount of fruits and vegetables, and increased consumption of legumes, nuts, and olive oil.

### Diet personalization

In both the EAT-IT and Control diet interventions, weight, height, and age measured at baseline (t0) were used to estimate Basal Metabolic Rate (BMR) using the Schofield equations. This BMR was then multiplied by a physical activity level (PAL) coefficient to estimate daily Total Energy Expenditure (TEE), as recommended by LARN [30]. A standardized PAL of 1.45 was applied. However, data from the IPAQ were also used to categorize the individual’s activity level as low, moderate, or high, allowing for adjustments to the PAL if necessary [31]. Finally, data from baseline food records were used to compare resulting energy intake and ensure that the dietary schemes were isocaloric with respect to their previous food intake and tailored as much as possible according to individual dietary habits and preferences. To adapt the EAT-IT dietary pattern, originally set for 2500 kcal, to different energy targets based on individual TEE, a specific Excel spreadsheet was developed. This tool allows for the adjustment of the energy associated with each food category in the pattern until the overall energy target is reached. It automatically generates the dietary scheme, reporting portion sizes and frequencies of consumption. This approach helps manage the different amounts included in the pattern, safeguarding nutritional adequacy. In this regard, food categories providing critical nutrients and present in reduced amounts (e.g., meat, dairy, and fish) were only slightly reduced or not reduced at all. In contrast, greater reductions were applied to food categories that were more abundant in the pattern and less commonly consumed by the reference population or individual subject (e.g., nuts and legumes in the Italian context). Although the reduction in food portions was not proportional across all categories, it still contributed to a further reduction in the corresponding environmental impact. Finally, maintaining appropriate consumption frequencies and realistic portion sizes was considered a practical constraint when developing individual dietary schemes.

### Anthropometric and body composition assessment

Anthropometric and body composition measurements were collected at baseline by trained personnel, at the International Center for the Assessment of Nutritional Status and the Development of Dietary Intervention Strategies (ICANS-DIS), following standardized international procedures [32]. Body weight was measured to the nearest 100 g with a column scale and with participants wearing only light underwear. Body height was measured to the nearest 0.1 cm using a vertical stadiometer. Body mass index (BMI) was calculated and classified according to NIH guidelines [33]. Waist (WC) and hip (HC) circumferences were measured with a non-stretch tape to the nearest 0.5 cm; WC at the midpoint between the last rib and the iliac crest, and HC at the level of the maximum circumference over the buttocks. Body composition was determined through plicometry. Skinfold thicknesses (biceps, triceps, subscapular, and suprailiac) were measured in triplicate using a Holtain caliper, and the mean value was used for analysis. Body density was then estimated using age- and sex-specific equations by Durnin and Womersley, and body fat percentage was derived using Siri’s formula [34, 35].

### Dietary habits and nutrient intake assessment

Before and after each intervention, participants were instructed to complete a 7-day weighed food record to assess their dietary habits and nutrient intake. The analysis of each food record was conducted using nutritional software (MetaDieta Professional 4.1.1, METEDA Srl, Rome, Italy), which allowed for the breakdown of food records into energy, macronutrient, and micronutrient intake.

### Feasibility and acceptability

To assess the feasibility and perceived acceptability of the EAT-IT dietary pattern, a validated questionnaire developed by Barnard and colleagues was administered at the end of each treatment [36]. This questionnaire was specifically designed to quantitatively evaluate the acceptability of a plant-based diet compared to a control diet in intervention studies. It assesses the ease of adherence to the intervention diet by considering various factors, such as ease of preparation, effort required, and adaptation to the diet. The questionnaire comprises eight questions: the first seven use a 7-point response scale, while the last question employs a 4-point scale. The questions were presented as follows: (1) How well do you like the current diet? (2) How easy is it to prepare? (3) Effort required by the diet? (4) How easy is it to continue this diet? (5) Which diet is easier to follow? (6) Acceptability of the EAT-IT dietary pattern? (7) In the future, I could stick with the EAT-IT dietary pattern. (8) Adapted to the EAT-IT dietary pattern? In general, more positive responses are associated with lower scores, while negative responses correspond to higher scores, except for questions 3 and 6, where the scoring is reversed. Questions 1–4 are applicable to both treatments, whereas questions 5–8 refer exclusively to the intervention diet. Consequently, participants completed the full questionnaire at the end of the EAT-IT intervention and an abbreviated version (questions 1–4) at the end of the control intervention.

### Biological samples collection and preparation

Before and after each dietary intervention, blood and fecal specimens were collected, as shown in Fig. 1.

Blood samples were obtained after an overnight fasting period using Vacutainer tubes containing ethylenediaminetetraacetic acid (EDTA) for whole blood collection, silicone gel for serum isolation, and trace element-free tubes for plasma collection to be used for trace metal assessment.

Whole blood samples were directly used for the assessment of complete blood count. Peripheral blood mononuclear cells (PBMCs) for the assessment of oxidative stress markers were isolated from whole blood using Histopaque 1077 density gradient centrifugation and cryopreserved in a medium consisting of 50% fetal bovine serum, 40% RPMI-1640, and 10% DMSO at − 80°C for later analysis [37].

Serum samples were obtained from blood to allow the assessment of biochemical and clinical markers (e.g., blood glucose and lipid profile), as well as inflammatory and oxidative stress markers quantified through ELISA kits. To obtain serum, blood samples were maintained at room temperature for a minimum of 30 min to allow clotting, followed by centrifugation at 1400 g for 15 min at 5 °C. The separated serum was then aliquoted and stored at − 80 °C until analysis.

Plasma was obtained from blood and subsequently used for the assessment of the exposure to essential and toxic metals. Whole blood was collected in tubes free from contaminants and immediately centrifuged at 1400 g for 15 min at 5 °C. The separated plasma was then aliquoted, transferred to contaminant-free collection tubes, and frozen at − 80 °C until analysis.

Fecal samples were used for the assessment of microbiota composition. Each participant was provided with sterile containers for the collection of fecal samples. Participants were instructed to store them in a cold environment (e.g., a refrigerator) immediately after collection and transport them to the laboratory within 24 h. If immediate transport was not possible, participants were requested to freeze the samples at − 20 °C to preserve the integrity of the microbiome.

### Biochemical and clinical markers

Biochemical markers were assessed in whole blood or in serum samples, including metabolic and functional parameters (i.e., complete blood count, glucose metabolism, lipid profile, liver, and renal function) analyzed by standardized validated protocol. In detail, complete blood count was performed using an automated hematology analyzer (ILAB 650, Instrumentation Laboratory, Lexington, MA, USA). This analysis included the measurement of white blood cells (WBCs), red blood cells (RBCs), hemoglobin, hematocrit, mean corpuscular volume (MCV), mean corpuscular hemoglobin (MCH), mean corpuscular hemoglobin concentration (MCHC), red cell distribution width (RDW), and platelet count, along with a differential count of neutrophils, lymphocytes, monocytes, eosinophils, and basophils.

Fasting glucose, insulin, triglycerides (TG), total cholesterol (TC), high-density lipoprotein cholesterol (HDL-C), aspartate aminotransferase (AST), alanine aminotransferase (ALT), gamma-glutamyl transferase (GGT), creatinine, and uric acid were quantified using a standardized automatic biochemical analyzer (Cobas 400, Roche Diagnostics, Basel, Switzerland, and Cobas e 411, Roche Diagnostics, Basel, Switzerland). Serum concentration of low-density lipoprotein cholesterol (LDL-C) was instead estimated by using the Friedewald formula [38]. Insulin sensitivity and β-cell function were assessed using the widely applied homeostasis model assessment (HOMA), based on fasting plasma glucose and insulin concentrations. Both the original model (HOMA1) and the updated computer model (HOMA2) were considered [39, 40]. For the computer model, the HOMA2 Calculator was used (version 2.2.3, Diabetes Trials Unit, University of Oxford).

### Inflammatory and oxidative stress markers

C-reactive protein was assessed in serum samples using a standardized automatic biochemical analyzer (ILAB 650, Instrumentation Laboratory, Lexington, MA, USA). Serum levels of tumor necrosis factor α (TNF-α) and interleukin-6 (IL-6), human lipopolysaccharide binding protein (LBP), adiponectin, and endothelin (EDN1) were evaluated by enzyme-linked immunosorbent assay (ELISA) kits: TNF-ɑ with BMS223HS, IL-6 with BMS213HS, LBP with EH297RB; adiponectin with BMS2032-2 (Invitrogen Corp, USA); EDN1 with EH0648 (Wuhan Fine Biotech Co., Ltd, Wuhan, Hubei, China).

Endogenous DNA damage, as a marker of oxidative stress, was assessed by the Comet Assay through an enzymatic treatment with formamidopyrimidine DNA glycosylase (FPG) performed on PBMCs previously isolated. All chemicals and reagents used for the analysis of DNA damage were obtained from Merck (Darmstadt, Germany). GelBond^®^ films were from VWR International S.r.l. (Monroeville, PA, USA), while the FPG enzyme was obtained by Norgenotech AS (Oslo, Norway). The analysis of DNA damage, particularly FPG-sensitive sites, was conducted using cryopreserved PBMCs. An aliquote of the PBMCs was gently thawed, washed, and resuspended in PBS for subsequent analysis. For the evaluation of FPG-sensitive sites, the prepared cell suspension was combined with LMP agarose and applied onto slides precoated with NMP agarose. This assay aimed to detect oxidized bases, focusing on 8-oxo-7,8-dihydro-2′-deoxyguanosine (8-oxodG) and ring-opened formamidopyrimidine nucleobases. The slides underwent lysis and subsequent treatment with FPG or buffer alone, serving as a control. Electrophoresis and subsequent analysis were conducted to migrate the DNA and determine the level of DNA damage. The nucleoid (i.e., the DNA that fills the cavity in the agarose where the whole cell was located prior to lysis) following the electrophoresis resembles a comet (hence the name of ‘comet assay’) with a circular head resulting from the undamaged DNA that remains in the cavity, and a tail constituted by the migrated damaged DNA. The brighter and longer the tail, the higher the level of damage. The assessment involved capturing and analyzing a hundred comets from each slide using specialized microscopy and image analysis software (Cometa 1.5; Immagini e Computer, Bareggio, Milan, Italy). The quantification of DNA damage was computed by subtracting the percentage of DNA in the tails of control cells from that in FPG-treated cells, providing insights into the extent of induced damage [37].

### Gut microbiota composition

Fecal samples were used to analyze gut microbiota composition. Gut microbiota was assessed through 16SrRNA gene quantification and taxonomic profiling. In brief, DNA was isolated from feces resuspended in Lysing Matrix E bead beating tubes (MPBio, Santa Ana, CA, USA) through the FastDNA™ SPIN Kit for Soil (MPBio) according to the manufacturer’s protocol. Then, the V3eV4 region of the 16 S rRNA gene was amplified with panbacterial primers16S341F (50eTCGTCGGCAGCGTCAGATGTGTATAAGAGACAGCCTACGGGNGGCWGCAGe30) and 16S806R (50eGTCTCGTGGGCTCGGAGATGTGTATAAGAGACAGGACTACHVGGGTATCTAATCCe30). Finally, amplicons were sequenced using a 600 cycle MiSeq v3 reagent kit (Illumina, San Diego, CA, USA). Subsequently, sequencing reads were subjected to pairing, filtering, taxonomic assignment, and biodiversity analyses by means of the bioinformatic pipeline Quantitative Insights Into Microbial Ecology (QIIME2) version 2023.2 through the Devisive Amplicon Denoising Algorithm (DADA2) using the Greengenes database (version 13_5). Illumina sequencing generated 4,030,722 filtered paired-end reads (median of 19,328 reads per sample). After merging and denoising by DADA2 the final sequences were 1,076,356 (mean = 5021, SD = 3306). The sequence length statistics in bp showed: min = 240, max = 457, median = 433, standard deviation = 25. Overall, 7729 unique amplicon sequence variants (ASVs) were identified.

### Metals in serum

Plasma samples collected in contaminant-free tubes was used to estimate dietary exposure to metals unintentionally added or naturally present in food such as selenium, manganese, cobalt, zinc, copper, nickel, aluminum, chromium, and cadmium. Metals were measured by inductively coupled plasma mass spectrometry (ICP-MS, 7850 Agilent) for the simultaneous determination of essential and non-essential metals in serum.

### Statistical analysis

Paired sample t-tests were conducted to evaluate within-group differences between baseline and post-treatment values. To assess the effect of the interventions, a general linear model (GLM) with repeated measures was applied, considering time (baseline and post-treatment) and treatment (EAT-IT and control) as independent variables. This approach aimed to test for significant time × treatment interactions, as well as the main effects of each variable. Statistical analyses were performed using SPSS software (Version 29.0.1.0; IBM Corp., Armonk, NY, USA). Additionally, taxonomic composition was analyzed using R software (Version 4.3.1; R Core Team, Vienna, Austria) by applying a non-parametric repeated measures ANOVA. Statistical significance was set at *p* < 0.05. Data are presented as mean ± standard deviation (SD) or as median and interquartile range (IQR; first to third quartile), depending on the distribution and type of variables. Outliers were identified using Tukey’s method, based on the interquartile range (IQR), and were excluded if they exceeded 1.5 times the IQR below the first quartile or above the third quartile. This procedure was performed prior to statistical analyses to enhance data robustness and minimize the potential bias introduced by extreme values. Data were analyzed on a per-protocol basis, meaning that participants who deviated from the study protocol, such as by missing required sessions or failing to follow standardized procedures, were excluded from the analysis. In case of missing data in one part of the intervention, data were entirely excluded from the analysis to minimize the risk of attrition and information bias.

## Results

### Baseline characteristics of the subjects

Overall, 10 subjects (4 men, 6 women) were enrolled in the pilot trial, but only 9 subjects completed the entire intervention, as one subject dropped out due to personal reasons. The main baseline characteristics of the 9 subjects who completed the study protocol are provided in Table 1. Participants were aged between 23 and 30 years, with an average age of 26. They had a high level of education, holding either a bachelor’s or master’s degree. Generally, they were moderately active, although there was considerable variability in physical activity levels. According to BMI values, participants were of normal weight. Overall, biochemical parameters indicated that the subjects were healthy.

**Table 1:** Baseline characteristics of subjects (*n* = 9) selected for the study

Variables	Mean ± SD
Age (years)	26 ± 2
Sex (n, %)	
Female	5 (56)
Male	4 (44)
Education (n, %)	
Basic education (primary)	0
Intermediate education (secondary)	0
High education (university level)	9 (100)
n° household component	2 ± 1
Total activity (MET-minutes/week)	3475 ± 2599
Weight Height (m)	62.4 ± 8.8 1.68 ± 0.10
BMI (kg/m2)	22.0 ± 1.4
Waist circumference (cm)	77.1 ± 6.2
Hip circumference (cm)	84.7 ± 5.7
Fat mass (%)	19.2 ± 6.1
Fat-free mass (%)	80.8 ± 6.1
Total cholesterol (mg/dL)	180 ± 36
HDL-cholesterol (mg/dL)	64 ± 18
LDL-cholesterol (mg/dL)	97 ± 35
TG (mg/dL)	151 ± 136
Fasting glycemia (mg/dL)	94 ± 7
Insulin (µU/mL)	9.1 ± 2.8
Creatinine (mg/dL)	0.9 ± 0.1
C-Reactive Protein (mg/L)	1.8 ± 2.7
ALT (U/L)	15.6 ± 5.0
AST (U/L)	17.5 ± 4.0
GGT (U/L)	19.2 ± 9.1
Uric acid (mg/dl)	5.2 ± 1.4
Urea (mg/dL) Haemoglobin - Hb (g/dL)	32 ± 6
Female	12.9 ± 0.9
Male	15.6 ± 0.6
Haematocrit (%)	
Female	40.9 ± 2.4
Male	45.9 ± 1.4
MCV (fL)	
Female	84.7 ± 3.6
Male	86.4 ± 2.2

All data are presented as mean ± standard deviation (SD) or as frequency (n, %), as appropriate. BMI, body mass index; MET, metabolic equivalent of task; HDL, high-density lipoprotein; LDL, low-density lipoprotein; TG, triglycerides; ALT, alanine aminotransferase; AST, aspartate aminotransferase; GGT, gamma-glutamyl transferase; Hb, hemoglobin; MCV, mean corpuscular volume

### Nutritional assessment

Data of nutrient intake is summarized in Table 2. The evaluation of food intake during the two intervention periods revealed differences in total energy and nutrient intake. Notable changes were observed in dietary fiber intake (from 11.3 ± 5.2 to 15.1 ± 4.6 g/1000 kcal for the EAT-IT intervention vs. from 11.0 ± 2.9 to 10.6 ± 3.1 g/1000 kcal for the Control intervention), omega-6 intake (from 5.7 ± 2.2 to 6.6 ± 2.7 g/day for the EAT-IT intervention vs. from 6.0 ± 2.3 to 5.0 ± 1.9 g/day for the Control intervention), and vitamin E intake (from 7.3 ± 3.5 to 9.7 ± 3.4 mg/day for the EAT-IT intervention vs. from 8.4 ± 3.7 to 7.2 ± 3.2 mg/day for the Control intervention). These changes represented the main differences between the two interventions (*p* < 0.05 for treatment*time interaction). Moreover, Control intervention resulted in a reduction (*p* < 0.05 for time) in total energy (from 1895 ± 380 kcal to 1648 ± 319 kcal) and carbohydrate intake (from 230.8 ± 51.1 g to 198.2 ± 38.1 g), with both changes being statistically significant (*p* < 0.05 for time).

**Table 2:** Energy and nutrient intake at the beginning and the end of the EAT-IT and control interventions

Dietary component	EAT-IT before	EAT-IT after	EAT-IT *p*-value (t)	Control diet before	Control diet after	Control diet *p*-value (t)	*p*-value (t x T)
Energy (kcal)	1913 ± 432	1810 ± 290	0.314	1895 ± 380	1648 ± 264	< 0.05*	0.272
Carbohydrate energy/total energy (%)	46.8 ± 4.9	48.1 ± 5.1	0.489	47.8 ± 3.2	47.6 ± 4.5	0.923	0.513
Sugar energy/total energy (%)	13.5 ± 1.9	14.5 ± 3.0	0.261	13.5 ± 2.2	14.3 ± 3.2	0.441	0.212
Protein energy/total energy (%)	16.8 ± 2.1	15.7 ± 1.3	0.177	16.1 ± 1.9	16.6 ± 0.9	0.406	0.107
Lipid energy/total energy (%)	35.9 ± 5.2	34.0 ± 4.1	0.342	35.2 ± 3.2	34.9 ± 4.8	0.752	0.440
Carbohydrates (g)	225.1 ± 51.1	225.1 ± 46.0	0.998	230.8 ± 51.1	198.2 ± 25.3	< 0.05*	0.163
Sugars (g)	63.6 ± 13.9	65.3 ± 15.6	0.788	63.1 ± 10.8	58.3 ± 14.5	0.364	0.426
Total fiber (g)	21.6 ± 10.7	27.2 ± 9.1	< 0.05*	20.4 ± 5.4	17.3 ± 5.4	0.155	< 0.01*
Fiber (g/1000 kcal)	11.3 ± 5.2	15.1 ± 4.2	< 0.01*	11.0 ± 2.9	10.6 ± 2.9	0.684	< 0.01*
Protein (g)	76.8 ± 16.4	67.9 ± 9.7	0.099	70.7 ± 8.6	65.5 ± 10.3	0.068	0.499
Lipids (g)	73.4 ± 23.0	66.0 ± 13.8	0.079	70.9 ± 17.6	61.9 ± 16.8	0.064	0.780
MUFA (g)	25.6 ± 12.1	28.4 ± 6.4	0.296	25.5 ± 12.5	21.8 ± 6.8	0.338	0.159
MUFA energy/total energy (%)	11.7 ± 3.6	14.5 ± 2.9	< 0.05*	12.6 ± 5.2	13.1 ± 5.1	0.686	0.212
PUFA (g)	8.4 ± 3.7	8.0 ± 2.3	0.705	7.4 ± 2.9	6.4 ± 1.7	0.208	0.668
PUFA energy/total energy (%)	3.9 ± 1.2	4.1 ± 1.0	0.775	3.6 ± 1.1	3.7 ± 0.9	0.925	0.831
SFA (g)	20.7 ± 8.6	16.7 ± 4.6	0.106	21.2 ± 6.3	18.0 ± 6.4	0.090	0.789
SFA energy/total energy (%)	13.0 ± 9.0	8.6 ± 1.5	0.187	18.1 ± 18.2	10.0 ± 2.1	0.216	0.595
ω-6 fatty acids (g)	5.7 ± 2.2	6.6 ± 1.9	0.214	6.0 ± 2.3	5.0 ± 1.3	0.11	< 0.05*
ω-6 energy/total energy (%)	2.7 ± 0.8	3.3 ± 0.9	0.127	2.9 ± 0.9	2.8 ± 0.6	0.62	0.100
ω-3 fatty acids (g)	1.2 ± 0.9	0.9 ± 0.3	0.383	0.7 ± 0.2	1.0 ± 0.6	0.19	0.149
ω-3 energy/total energy (%)	0.6 ± 0.4	0.5 ± 0.1	0.475	0.4 ± 0.1	0.6 ± 0.3	0.07	0.094
Cholesterol (mg)	157.0 ± 94.8	124.3 ± 51.3	0.135	166.9 ± 59.6	147.7 ± 68.1	0.62	0.752

All data are expressed as mean ± standard deviation (SD); t x T: time x treatment; SFA, saturated fatty acids; MUFA, monounsaturated fatty acids; PUFA, polyunsaturated fatty acids; ω-6, omega-6 fatty acids; ω-3, omega-3 fatty acids. * indicates that the difference between interventions is statistically significant considering *p* < 0.05

### Feasibility and acceptability

**Fig. 2:**
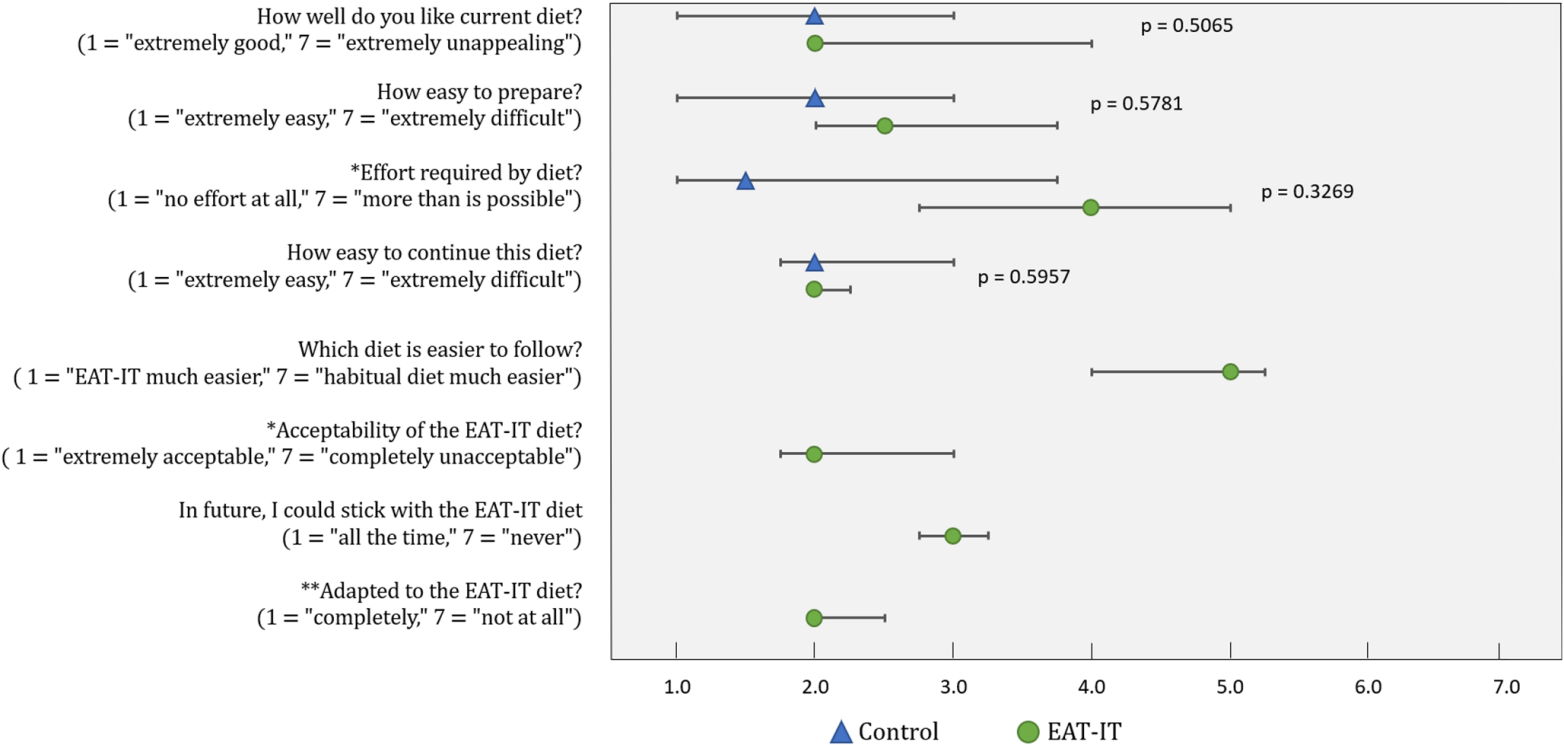
Feasibility of the EAT-IT dietary pattern compared to the Control one, according to the validated questionnaire of Barnard et al. [36]. All data are expressed as median. (SD). The horizontal bars represent the interquartile range between the first and third quartiles. The questions are presented in a simplified form compared to the full version actually included in the questionnaire administered to the participants. * indicates that the score was reversed to maintain the orientation of positive scores on the left and negative scores on the right, even if the question originally had inverted scoring. ** indicates that the scores were rescaled to a 1–7 scale to allow comparability with other questions, as the original question used a 1–4 scale. p-values were calculated using independent-samples t-tests only for the first four questions, as the last four questions were not applicable to the control group

Data from the administered questionnaire and direct feedback from the volunteers at the end of the study indicated an overall positive response, although some critical issues also emerged. The median scores from the validated questionnaire are reported in Fig. 2, where positive scores are oriented towards the left and negative scores towards the right. Questions 1–4 compared various aspects of diet acceptability between the EAT-IT dietary pattern and the Control. No statistical differences were found in the collected responses; however, the answer to question 3, which relates to the perceived effort of the intervention, revealed a less positive score for the EAT-IT dietary pattern. Question 5 asked participants to compare the ease of following the EAT-IT dietary pattern with their habitual diet. Responses indicated that participants generally found their habitual diet easier to follow.

Questions 6, 7, and 8 required participants to evaluate overall acceptability, willingness to continue with the diet, and levels of adaptation respectively, exclusively considering the EAT-IT dietary pattern. Questions 6 and 8 indicated good levels of acceptability, whereas question 7 showed only a mild willingness to stick with the EAT-IT dietary pattern. Based on direct feedback, volunteers verbally reported that the diet was ‘sufficiently good’ and tolerable in terms of preparation, even dough also challenging due to legumes preparation. For the same reason, one participant evaluated this pattern as ‘extremely difficult to prepare”. Reducing meat and dairy intake was generally found easier than increasing legume and whole-grain consumption.

### Biochemical parameters

Data on the effect of the EAT-IT intervention versus the Control on biochemical parameters are presented in Table 3. No significant changes were observed within or between groups, except for insulin, which decreased following the EAT-IT intervention (*p* < 0.05, within-group analysis), along with HOMA1-IR and HOMA2-IR. Although not statistically significant, a trend toward cholesterol reduction was also observed after the EAT-IT intervention (*p* = 0.066).

**Table 3:** Effects of interventions on biochemical markers

Biochemical parameters	EAT-IT before	EAT-IT after	EAT-IT *p*-value (t)	Control diet before	Control diet after	Control diet *p*-value (t)	*p*-value (t x T)
Fasting Glycemia (mg/dL)	95 ± 8	91 ± 6	0.161	95 ± 6	91 ± 6	0.145	0.974
Insulin (µU/mL)	8.4 ± 2.2	6.5 ± 2.2	< 0.05*	8.8 ± 3.4	8.3 ± 3.2	0.698	0.344
HOMA1-IR	2.0 ± 0.6	1.5 ± 0.5	< 0.05*	2.1 ± 0.9	1.9 ± 0.8	0.556	0.401
HOMA2-IR	1.1 ± 0.3	0.9 ± 0.3	< 0.05*	1.1 ± 0.5	1.1 ± 0.4	0.748	0.298
HOMA1-%β	98.1 ± 30.9	86.9 ± 37.7	0.364	98.6 ± 31.3	105.2 ± 35.1	0.636	0.332
HOMA2-%β	91.3 ± 19.1	83.0 ± 22.1	0.287	90.5 ± 20.7	95.7 ± 21.6	0.547	0.239
Total Cholesterol (mg/dL)	181 ± 42	166 ± 39	0.066	177 ± 38	176 ± 28	0.894	0.185
HDL (mg/dL)	63 ± 17	59 ± 14	0.250	65 ± 16	62 ± 17	0.338	0.961
LDL (mg/dL)	100 ± 39	94 ± 35	0.252	101 ± 35	97 ± 29	0.370	0.811
TG (mg/dL)	100 ± 35	106 ± 46	0.686	105 ± 43	124 ± 59	0.181	0.527
Uric Acid (mg/dL)	5.3 ± 1.2	5.8 ± 1.6	0.172	5.4 ± 1.8	5.5 ± 1.4	0.858	0.382
Creatinine (mg/dL)	0.9 ± 0.1	0.8 ± 0.1	0.447	0.9 ± 0.1	0.8 ± 0.1	0.347	0.715
ALT (U/L)	17.6 ± 10.3	15.6 ± 5.3	0.510	14.7 ± 4.7	16.1 ± 8.8	0.549	0.367
AST (U/L)	19.8 ± 10.3	18.8 ± 4.3	0.703	16.6 ± 3.5	16.6 ± 4.5	0.980	0.720
GGT (U/L)	20.2 ± 9.2	21.9 ± 12.6	0.350	20.3 ± 11.5	20.9 ± 12.3	0.608	0.596

All data are expressed as mean ± standard deviation (SD). t x T: time x treatment; AST, Aspartate Aminotransferase; ALT, Alanine Aminotransferase; GGT, Gamma-Glutamyl Transpeptidase; HOMA1-IR, Homeostasis Model Assessment for Insulin Resistance derived from the original model; HOMA2-IR, Homeostasis Model Assessment for Insulin Resistance calculated using the updated computer model; HOMA1-%β, Homeostasis Model Assessment for Beta-Cell Function derived from the original model; HOMA2-%β, Homeostasis Model Assessment for Beta-Cell Function calculated using the updated computer model; HDL, High-Density Lipoprotein; LDL, Low-Density Lipoprotein; TG, Triglycerides. * indicates that the difference between interventions is statistically significant considering *p* < 0.05

Differences within time and treatment (time*Treatment) interaction are reported

### Inflammatory and oxidative stress markers

Regarding oxidative stress markers, following the EAT-IT intervention a significant reduction in net-FPG sensitive sites (% DNA in tail) was observed, while inflammatory markers did not change significantly after the EAT-IT and Control diets (see Table 4).

**Table 4:** Effect of interventions on inflammatory and oxidative stress markers

Inflammatory and oxidative stress markers	EAT-IT before	EAT-IT after	EAT-IT *p*-value (t)	Control diet before	Control diet after	Control diet *p*-value (t)	*p*-value (t x T)
Adiponectin (µg/mL)	13.3 ± 5.1	10.7 ± 4.7	0.056	12.6 ± 4.8	12.3 ± 5.0	0.689	0.130
C-Reactive Protein (mg/L)	1.8 ± 2.8	0.7 ± 1.0	0.141	0.7 ± 0.7	0.9 ± 1.2	0.557	0.104
Endothelin-1 (pg/mL)	0.4 ± 0.2	0.4 ± 0.2	0.748	0.4 ± 0.2	0.4 ± 0.2	0.729	0.633
IL-6 (pg/mL)	0.5 ± 0.4	0.5 ± 0.5	0.981	0.7 ± 1.0	0.4 ± 0.3	0.327	0.360
LBP (mg/L)	47.2 ± 16.8	34.0 ± 9.1	0.076	39.8 ± 5.1	40.0 ± 7.5	0.964	0.099
TNF-α (pg/mL)	2.5 ± 1.8	2.3 ± 0.9	0.810	1.8 ± 0.9	2.3 ± 2.1	0.417	0.416
Net-FPG sensitive sites (% DNA in tail)	17.5 ± 9.3	8.5 ± 4.1	< 0.05*	16.5 ± 13.0	13.8 ± 9.7	0.308	0.155

All data are expressed as mean ± standard deviation (SD). t x T, time x treatment interaction; FPG, Formamidopyrimidine DNA Glycosylase; IL-6, Interleukin-6; LBP, Lipopolysaccharide-Binding Protein; TNF-α, Tumour necrosis factor-alpha. * indicates that the difference between interventions is statistically significant considering *p* < 0.05

### Gut microbiota composition

**Fig. 3:**
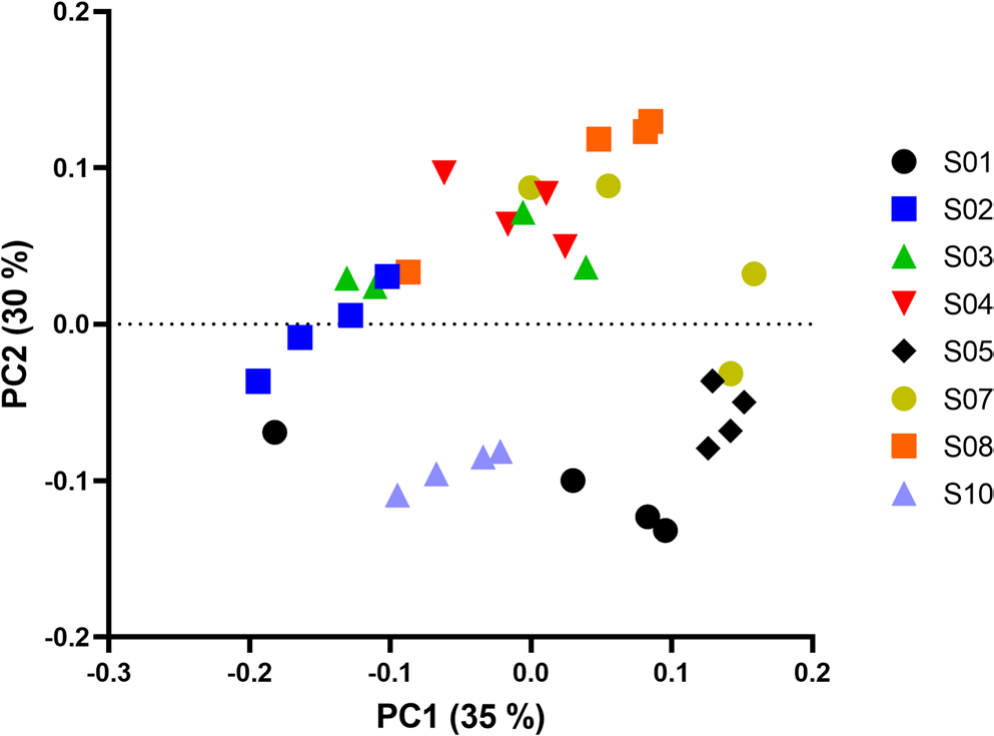
Principal coordinates analysis of weighted UniFrac distances. Evaluation of any pre- and post-treatment differences within the group on gut microbiota composition

Regarding the diversity of the gut microbiota across treatments, the study found no significant differences in alpha diversity indices, including both richness and evenness (data not shown). β-diversity was also highly variable, with greater variability observed between subjects than within the same subject following the interventions, as illustrated in Fig. 3.

**Fig. 4:**
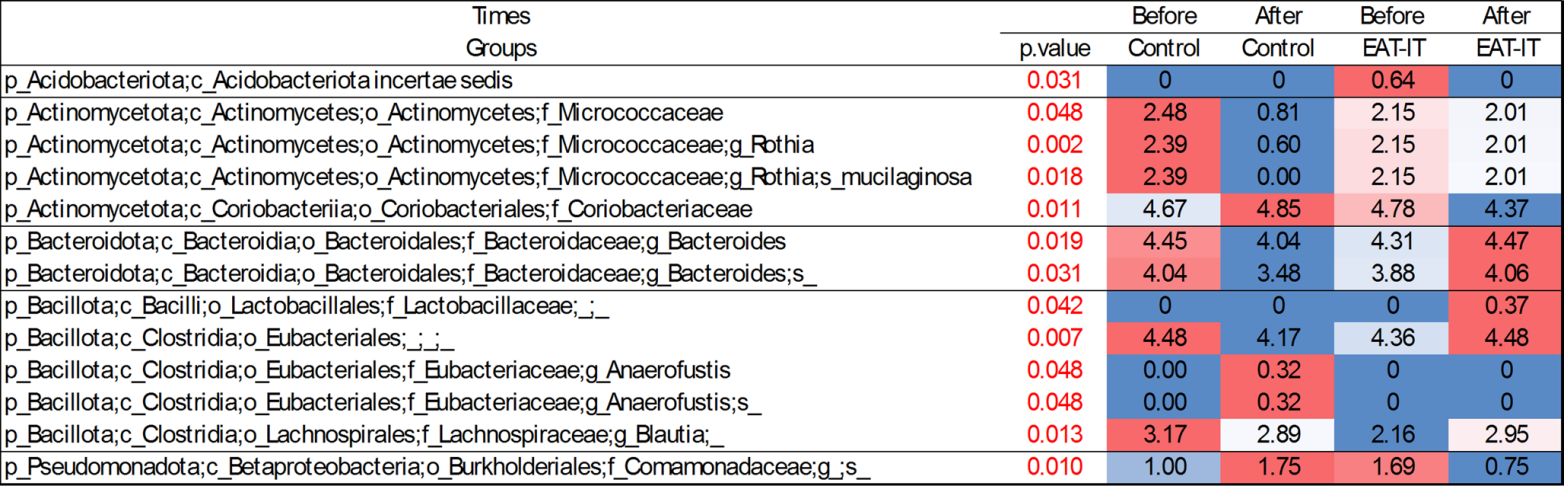
Non-parametric repeated measures ANOVA of bacterial taxa in fecal samples. The red and blue highlights indicate taxa with median values representing high and low presence in the fecal samples, respectively

Differences between treatments were primarily observed in the taxonomic composition. Specifically, there was a decrease in the abundance of *Rothia mucilaginosa*, the genus *Blautia*, and a species within the family Comamonadaceae. Additionally, the entire Coriobacteriaceae family showed a reduction following EAT-IT intervention. Conversely, several taxa exhibited an increase after EAT-IT intervention, including the genus *Bacteroides* and a specific species within this genus, the Lactobacillaceae family, and an unidentified species within the order Clostridiales, as depicted in Fig. 4.

### Metals in serum

Levels of serum selenium, manganese, cobalt, zinc, copper, nickel, aluminum, chromium, and cadmium were not different after the EAT-IT when compared to the Control treatment (data not shown). On the other hand, chromium levels decreased significantly following both the EAT-IT (from 0.56 ± 0.11 µg/L to 0.44 ± 0.16 µg/L, *p* < 0.05) and the Control treatment (from 0.60 ± 0.17 µg/L to 0.39 ± 0.25 µg/L, *p* < 0.01). Also, a tendency towards lower zinc and manganese levels were found after the Control diet, while aluminum levels decreased significantly following the EAT-IT intervention (from 2.77 ± 0.76 µg/L to 1.84 ± 1.00 µg/L, *p* < 0.01).

## Discussion

The present pilot study explored both the feasibility and effect on health-related parameters of a previously developed healthy and sustainable dietary pattern modeled on the ELHRD and tailored for the Italian population. To our knowledge, this is the first dietary intervention study directly testing an ELHRD-based diet [41]. Overall, results showed promising benefits in terms of nutritional and metabolic status (e.g., increased fiber intake and insulin response), alongside potential critical aspects that warrant further investigation.

Given the theoretical nature of the EAT-IT dietary pattern, this pilot study represents a preliminary step toward its practical implementation and was conducted in a small sample of young, healthy Italian subjects.

In this regard, the EAT-IT dietary pattern includes the consumption of a high amount of legumes and nuts, in line with the ELHRD [22, 26]. In Italy, as well as in several other areas of the world, the consumption of these food categories is lower than the current recommendations, which are still lower compared to the quantities required by the EAT-IT dietary pattern [29, 42]. Legume acceptability represents a key issue, for their consumption is often hindered by factors such as gastrointestinal discomfort and time-consuming preparation [42–44]. Common barriers regarding legumes consumption include flatulence, difficulty in preparation, lack of knowledge on incorporating legumes into meals, and a perception of legumes as vegetarian food [44–46]. In our study, most participants reported moderate to high appreciation for the overall diet’s taste, while reporting difficulties mainly related to legume preparation, which interestingly was found to be a more difficult task than reducing milk and meat. Therefore, addressing these barriers is essential for the successful promotion of sustainable dietary patterns in real life-scenarios [47, 48].

Based on food records data, significant changes were observed following the EAT-IT intervention, including increases in total fiber, energy-standardized fiber intake (g/1000 kcal), MUFAs, while energy intake decreased after the Control intervention. Notably, total fiber intake was significantly higher at the end of the EAT-IT intervention compared to the final values observed after the Control intervention. After the EAT-IT intervention, fiber intake aligned with Italian recommendations (12.6–16.7 g/1000 kcal) and reached the suggested dietary target of 25 g/day [30].

However, despite these improvements, fiber intake at the end of the EAT-IT intervention did not approach the theoretical intake calculated for this dietary pattern, which was approximately 17.5 g/1000 kcal [26]. Furthermore, baseline fiber consumption was below Italian recommendations, with values of 21.6 ± 10.7 g (11.3 ± 5.2 g/1000 kcal) prior to the EAT-IT intervention and 20.4 ± 5.4 g (11.0 ± 2.9 g/1000 kcal) prior the Control intervention.

Numerous studies have demonstrated that a high intake of dietary fibers in adults is associated with various health benefits, including improvements in lipid profile and cardiovascular health, reduced incidence of some gastrointestinal diseases, a lower risk of cancer (particularly colorectal cancer), as well as an overall decrease in all-cause mortality [49–52]. However, current Western diets are often reported to be low in dietary fiber [53]. Available data indicate that fiber intake among Italian adults (18–64 years) is approximately 18.7 g/day, similar to values reported in other European and non-European countries, including USA (18.3 g/day) and Japan (18.3 g/day) [53, 54]. Even in countries with relatively higher intakes, such as Germany and Norway, average fiber consumption rarely exceeds 25 g/day [53].

Regarding fats, total lipid intake showed a decreasing trend across both dietary patterns, while a significant increase in the proportion of energy derived from MUFAs was observed following the EAT-IT intervention. Additionally, a significant time × treatment interaction was observed for ω-6 fatty acids intake between the EAT-IT and Control interventions. This data reflects the high amount of nuts included in the EAT-IT dietary pattern, although the final values remained lower than expected, even after adjusting for the energy intake observed at the end of the intervention [26]. This suggests that nut consumption, although not explicitly highlighted in participants’ feedback, may have represented an additional challenge in adhering to the dietary pattern.

Regarding total lipid intake, it is important to note that energy derived from lipids was above the upper range of the reference intake of 35% of total energy at baseline for both interventions. Even after the observed reduction, it remained only slightly below this threshold. While final values after the EAT-IT intervention approached the theoretical intake (36.2%), lipid intake following the Control intervention remained higher than expected (i.e., 30.3%) [26].

Finally, although both treatments resulted in a decrease in total energy intake, a statistically significant decrease (approximately 250 kcal) was observed only after the Control intervention. This reduction was mainly driven by lower consumption of carbohydrate-rich foods, and to a lesser extent by reduced lipid intake, possibly reflecting circumstantial factors unrelated to the intervention or difficulties in implementing the provided dietary guidelines, which should be further explored.

Regarding the impact of the two dietary interventions on the biomarkers under study, we observed a significant reduction in fasting plasma insulin concentration over time following the EAT-IT dietary pattern. Notably, this was accompanied by a reduction in both HOMA1-IR and HOMA2-IR, surrogate markers of insulin resistance derived from insulin and glucose concentrations. HOMA-IR is a well-established measure of insulin resistance that has been extensively validated through comparisons with other models, clinical assessments, and epidemiological data [40, 55]. A healthy plant-based diet typically includes legumes, whole grains, fruits, vegetables, and nuts, whose intake is generally associated with improved glucose metabolism and a reduced risk of developing type 2 diabetes [56–59]. Interestingly, we also observed a reduction in HOMA1-%β, although not statistically significant, following the EAT-IT intervention. This might be mistakenly interpreted as a decline in β-cell function; however, as highlighted in the literature on the appropriate use of these indices, an increase in insulin sensitivity in healthy individuals is often accompanied by reduced insulin secretion from β-cells [40, 60].

Furthermore, our study showed a trend toward reduced serum LBP, a marker of intestinal permeability [61]. LBP, produced by the liver, rises in response to LPS derived from the outer membrane of Gram‑negative bacteria. In the presence of increased intestinal permeability, LPS may enter the bloodstream and trigger inflammatory responses, via Toll-like receptor 4. Consequently, LBP is commonly used as a surrogate marker for circulating LPS in human studies. Previous evidence has shown a negative association between vegetable intake and plasma LBP concentration [62], and prebiotic fibers and polyphenols have been suggested to reduce circulating LPS levels [62, 63].

Despite this trend of reduction in LBP, in our intervention study inflammatory parameters did not change. Healthy plant-based diets are reported to positively modulate inflammatory biomarkers [64]. However, the characteristics of the investigated dietary patterns and the study population can largely affect the results of such interventions [61, 64]. Most studies have been carried out on participants with overweight or obesity, or chronic diseases, while little is known about the effects on healthy subjects [61]. Our findings are in line with those of Pražnikar et al., (2023) who did not find significant changes in inflammatory parameters after omnivore, vegan and vegetarian dietary interventions in apparently healthy adults [61]. They concluded that in healthy, non-obese individuals it is more difficult to appreciate the impact of diet on inflammatory status given the complex interplay of dietary and non-dietary factors involved in its regulation.

Intriguingly, the EAT-IT diet significantly lowered DNA damage as marker of oxidative stress. Similar findings have been reported for the Italian population with the consumption of various plant-based foods. Specifically, studies on broccoli, tomatoes, hazelnuts, and blueberries have demonstrated positive effects on reducing DNA damage evaluated by the Comet assay. For instance, a study observed a significant reduction in H_2_O_2_-induced DNA damage in blood mononuclear cells following 10 days of broccoli consumption in smokers and non-smokers [65]. Drinking tomato juice for 26 days significantly reduced lymphocyte DNA damage [66]. Hazelnuts were shown to significantly decrease endogenous DNA damage and oxidatively induced DNA strand breaks after 8 weeks of consumption in children with primary hyperlipidemia [67]. Additionally, blueberries significantly reduced H₂O₂-induced DNA damage one hour after consumption, compared to the control group [68]. These findings support the protective role of diets rich in micronutrients and bioactive compounds against oxidative DNA damage [69].

This study also highlighted changes in microbiota composition following the EAT-IT diet. One of the most notable changes in microbial composition was the increase in the genus *Bacteroides*. This genus is often associated with a diet high in fiber and complex polysaccharides, such as those found in legumes. *Bacteroides* species have the enzymatic capability to degrade these complex carbohydrates, leading to their proliferation in response to a legume-rich diet. This observation is consistent with previous evidence indicating dietary fiber as a key driver of gut microbiota composition [70].

Conversely, the decrease in *Rothia mucilaginosa* and members of the Coriobacteriaceae family observed after the EAT-IT diet could reflect the shift in the gut environment caused by the increased intake of fermentable fibers. These fibers are preferentially utilized by other microbial groups, potentially outcompeting *Rothia* and Coriobacteriaceae, which might not be as adept at fiber degradation. This finding is in line with studies showing that high-fiber diets selectively promote fiber-degrading bacteria while reducing the abundance of other taxa [71]. Some members of Coriobacteriaceae are involved in the metabolism of steroids and bile acids. These processes can lead to the production of metabolites that, in excess, may contribute to inflammatory conditions or increase the risk of metabolic diseases such as metabolic syndrome or certain cardiovascular diseases [72]. Overall, these results further highlight the role of diet in modulating gut microbiota composition and suggest that the beneficial effects of the EAT-IT dietary pattern may be partly mediated by microbiota-related mechanisms, including colonic fermentation and short-chain fatty acids production, which are known to influence glucose metabolism and energy homeostasis [73].

The FAO specifies that one of the indispensable elements for a diet to be considered healthy and sustainable is food safety [28]. Food contamination with toxic substances such as metals (e.g., lead, cadmium, mercury, and arsenic), mycotoxins and processing contaminants (e.g., acrylamide) can occur at any stage in the food chain, potentially leading to adverse effect on the environment and human health in case the exposure exceeds certain levels [74–76]. To avoid harmful exposure, international health authorities and governments closely monitor the levels of heavy metals in food, establish safety limits to protect public health, and sets maximum levels for certain contaminants in food products [77, 78]. As a result, dietary intake of harmful metals is generally lower than health-based guidance values [77, 79]. Our analysis of serum levels of essential and non-essential metals such as selenium, manganese, cobalt, zinc, copper, nickel, aluminum, chromium, and cadmium showed that these levels within reference values normally found in the general population [80]. Among the metals studied, cadmium is of particular concern due to its harmful effects over time and EFSA reported algae to be one of the main critical products, however such products were not consumed by our volunteers [75]. In our study we recorded a cadmium level of 0.16 µg/L, which falls within the normal range. Nickel is also noteworthy, as legumes and nuts are significant sources of nickel [81]. Despite EFSA concerns regarding long-term dietary nickel exposure, the average serum nickel concentration observed in our study was consistent with exposure levels reported in previous study [82]. Our results do not suggest additional exposure to toxic metals when transitioning to a plant-based diet rich in grains, legumes, and nuts.

Overall, this pilot study provides exploratory evaluation of key dimensions of diet sustainability, including nutritional adequacy, acceptability, and selected metabolic and health outcomes. As a pilot study, the main limitations include the small sample size and the lack of detailed micronutrient intake data. Nevertheless, the insights gained from this initial assessment of a healthy population in conditions minimizing potential confounding factors will inform further refinement of the EAT-IT dietary pattern and support its validation in larger and more representative cohorts. In this context, future studies should place greater emphasis on micronutrient adequacy, particularly for nutrients such as vitamin B12 and iron, which have been reported as potentially critical in ELHRD-based and low-environmental-impact dietary patterns [23, 83].

## Conclusions

This pilot study represents a first attempt to advance the understanding of the overall impact of sustainable dietary patterns on acceptability, as well as on metabolic and functional health-related outcomes, by providing important insights into numerous interconnected factors in a real-life setting. This study highlighted potential challenges associated with the quantity and frequency of the provided plant-based foods, particularly regarding their acceptability among subjects who do not regularly consume them. Moving forward, these findings will help inform the design of larger study enabling a more comprehensive understanding of all the facets involved in establishing a long-term dietary pattern that can seamlessly integrate into everyday life.

## Data Availability

Data will be made available on request.

## Abbreviations

ALT: Alanine aminotransferase
ASVs: Amplicon sequence variants
AST: Aspartate aminotransferase
BMI: Body mass index
BMR: Basal metabolic rate
DADA2: Devisive amplicon denoising algorithm
EAT-IT: Italian-mediterranean dietary pattern developed based on the EAT-Lancet Reference Diet
EDN1: Endothelin
EDTA: Ethylenediaminetetraacetic acid
ELHRD: EAT-lancet commission healthy reference diet
FBDGs: Food-based dietary guidelines (Italian)
FPG: Formamidopyrimidine DNA Glycosylase
GGT: Gamma-glutamyl transferase
Hb: Hemoglobin
HDL: High-density lipoprotein
HDL-C: High-density lipoprotein cholesterol
HOMA: Homeostasis model assessment
HOMA1-%β: Homeostasis model assessment for beta-cell function derived from the original model
HOMA2-%β: Homeostasis model assessment for beta-cell function calculated using the updated computer model
HOMA1-IR: Homeostasis model assessment for insulin resistance derived from the original model
HOMA2-IR: Homeostasis model assessment for insulin resistance calculated using the updated computer model
IL-6: Interleukin-6
IPAQ: International physical activity questionnaire
IQR: Interquartile range
LARN: Livelli di assunzione di riferimento di nutrienti ed energia (Reference Intake Levels of Nutrients and Energy for the Italian Population)
LBP: Lipopolysaccharide-binding protein
LDL: Low-density lipoprotein
LDL-C: Low-density lipoprotein cholesterol
LPS: Lipopolysaccharide
MCH: Mean corpuscular hemoglobin
MCHC: Mean corpuscular hemoglobin concentration
MCV: Mean corpuscular volume
MET: Metabolic equivalent of task
PAL: Physical activity level
PBMCs: Peripheral blood mononuclear cells
QIIME2: Quantitative Insights Into Microbial Ecology
RBCs: Red blood cells
RDW: Red cell distribution width
TC: Total cholesterol
TEE: Total energy expenditure
TG: Triglycerides
TNF-α: Tumor necrosis factorα
WBCs: White blood cells

## References

[1] Miller V, Webb P, Cudhea F et al (2022) Global dietary quality in 185 countries from 1990 to 2018 show wide differences by nation, age, education, and urbanicity. Nat Food 3:694–702. 10.1038/s43016-022-00594-9 37118151 10.1038/s43016-022-00594-9 PMC10277807

[2] Mertens E, Kuijsten A, Dofková M et al (2019) Geographic and socioeconomic diversity of food and nutrient intakes: a comparison of four European countries. Eur J Nutr 58:1475–1493. 10.1007/s00394-018-1673-6 29594476 10.1007/s00394-018-1673-6 PMC6561990

[3] Afshin A, Sur PJ, Fay KA et al (2019) Health effects of dietary risks in 195 countries, 1990–2017: a systematic analysis for the Global Burden of Disease Study 2017. Lancet 393:1958–1972. 10.1016/S0140-6736(19)30041-8 30954305 10.1016/S0140-6736(19)30041-8 PMC6899507

[4] Hemler EC, Hu FB (2019) Plant-based diets for personal, population, and planetary health. Adv Nutr 10:S275–S283. 10.1093/advances/nmy117 31728495 10.1093/advances/nmy117 PMC6855934

[5] Doré J, Blottière H (2015) The influence of diet on the gut microbiota and its consequences for health. Curr Opin Biotechnol 32:195–199. 10.1016/j.copbio.2015.01.002 25615931 10.1016/j.copbio.2015.01.002

[6] Trautwein EA, McKay S (2020) The role of specific components of a plant-based diet in management of dyslipidemia and the impact on cardiovascular risk. Nutrients 12:2671. 10.3390/nu12092671 32883047 10.3390/nu12092671 PMC7551487

[7] Liu RH (2013) Health-promoting components of fruits and vegetables in the diet. Adv Nutr 4. 10.3945/an.112.003517. :384S-392S 10.3945/an.112.003517 PMC3650511 23674808

[8] Slavin JL, Lloyd B (2012) Health benefits of fruits and vegetables. Adv Nutr 3:506–516. 10.3945/an.112.002154 22797986 10.3945/an.112.002154 PMC3649719

[9] Martini D (2019) Health benefits of Mediterranean diet. Nutrients 11:1802. 10.3390/nu11081802 31387226 10.3390/nu11081802 PMC6723598

[10] Sidhu SRK, Kok CW, Kunasegaran T, Ramadas A (2023) Effect of plant-based diets on gut microbiota: a systematic review of interventional studies. Nutrients 15:1510. 10.3390/nu15061510 36986240 10.3390/nu15061510 PMC10057430

[11] Medawar E, Huhn S, Villringer A, Veronica Witte A (2019) The effects of plant-based diets on the body and the brain: a systematic review. Transl Psychiatry 9:226. 10.1038/s41398-019-0552-0 31515473 10.1038/s41398-019-0552-0 PMC6742661

[12] Poore J, Nemecek T (2018) Reducing food’s environmental impacts through producers and consumers. Sci (1979) 360:987–992. 10.1126/science.aaq0216 10.1126/science.aaq0216 29853680

[13] Jalava M, Kummu M, Porkka M et al (2014) Diet change—a solution to reduce water use? Environ Res Lett 9:074016. 10.1088/1748-9326/9/7/074016

[14] Gibbs J, Cappuccio FP (2022) Plant-based dietary patterns for human and planetary health. Nutrients 14:1614. 10.3390/nu14081614 35458176 10.3390/nu14081614 PMC9024616

[15] Springmann M, Wiebe K, Mason-D’Croz D et al (2018) Health and nutritional aspects of sustainable diet strategies and their association with environmental impacts: a global modelling analysis with country-level detail. Lancet Planet Health 2:e451–e461. 10.1016/S2542-5196(18)30206-7 30318102 10.1016/S2542-5196(18)30206-7 PMC6182055

[16] Moreno LA, Meyer R, Donovan SM et al (2022) Perspective: striking a balance between planetary and human health—is there a path forward? Adv Nutr 13:355–375. 10.1093/advances/nmab139 34849542 10.1093/advances/nmab139 PMC8970843

[17] McLaren S, Berardy A, Henderson A, Holden N, Huppertz T, Jolliet O, De Camillis C, Renouf M, Rugani B, Saarinen M, van der Pols J, Vázquez-Rowe I, Antón Vallejo A, Bianchi M, Chaudhary A, Chen C, Cooreman Algoed M, Dong H, Grant T, Green A, Hallström E, Hoang H, Leip A, Lynch J, McAuliffe G, Ridoutt B, Saget S, Scherer L, Tuomisto H, Tyedmers P, van Zanten H (2021) Integration of environment and nutrition in life cycle assessment of food items: opportunities and challenges. FAO, Rome. 10.4060/cb8054en

[18] Martini D, Tucci M, Bradfield J et al (2021) Principles of sustainable healthy diets in worldwide dietary guidelines: efforts so far and future perspectives. Nutrients 13(6):1827. 10.3390/nu13061827 34071933 10.3390/nu13061827 PMC8228140

[19] FAO and WHO (2019) Sustainable healthy diets – Guiding Principles. Rome

[20] CREA. Linee Guida per una Sana Alimentazione; CREA: Rome, Italy (2018) ; pp. 1–231

[21] HLPE (2020) Food security and nutrition: building a global narrative towards 2030. a report by the high level panel of experts on food security and nutrition of the committee. on World Food Security (Rome)

[22] Willett W, Rockström J, Loken B et al (2019) Food in the Anthropocene: the EAT–Lancet Commission on healthy diets from sustainable food systems. Lancet 393:447–492. 10.1016/S0140-6736(18)31788-4 30660336 10.1016/S0140-6736(18)31788-4

[23] Leonard UM, Leydon CL, Arranz E, Kiely ME (2024) Impact of consuming an environmentally protective diet on micronutrients: a systematic literature review. Am J Clin Nutr 119:927–948. 10.1016/j.ajcnut.2024.01.014 38569787 10.1016/j.ajcnut.2024.01.014

[24] FAO (2023) Contribution of terrestrial animal source food to healthy diets for improved nutrition and health outcomes – an evidence and policy overview on the state of knowledge and gaps. FAO, Rome. 10.4060/cc3912en

[25] Tso R, Forde CG (2021) Unintended consequences: nutritional impact and potential pitfalls of switching from animal- to plant-based foods. Nutrients 13:2527. 10.3390/nu13082527 34444686 10.3390/nu13082527 PMC8398225

[26] Tucci M, Martini D, Del Bo’ C et al (2021) An Italian-Mediterranean dietary pattern developed based on the EAT-Lancet Reference Diet (EAT-IT): a nutritional evaluation. Foods 10:558. 10.3390/foods10030558 33800396 10.3390/foods10030558 PMC8002105

[27] Tucci M, Martini D, Marino M et al (2022) The environmental impact of an Italian-Mediterranean dietary pattern based on the EAT-Lancet reference diet (EAT-IT). Foods 11:3352. 10.3390/foods11213352 36359965 10.3390/foods11213352 PMC9658895

[28] FAO. Sustainable diet and biodiversity; FAO: Rome, Italy (2010) ; pp. 1–309

[29] Rossi L, Ferrari M, Ghiselli A (2023) The Alignment of recommendations of dietary guidelines with sustainability aspects: lessons learned from Italy’s Example and Proposals for Future Development. Nutrients 15:542. 10.3390/nu15030542 36771249 10.3390/nu15030542 PMC9921064

[30] SINU (2014) LARN - Livelli di assunzione di riferimento di nutrienti ed energia - IV Revisione. Società Italiana di Nutrizione Umana, SINU, Milano

[31] Craig WJ (2010) Nutrition concerns and health effects of vegetarian diets. Nutr Clin Pract 25:613–620. 10.1177/0884533610385707 21139125 10.1177/0884533610385707

[32] Lohman TG, Roche AF, Martorell R (1988) Anthropometric standardization reference manual. Human Kinetics Books, Champaign, IL

[33] National Institutes of Health (1998) Executive Summary. 10.1002/j.1550-8528.1998.tb00690.x. Obes Res 6:

[34] Siri WE (1961) In: Brozek J, Henschel A (eds) Body composition from fluid spaces and density: analysis of methods. Techniques for measuring body compositions, National Academy of Sciences, Washington DC, p 233

[35] Durnin JVGA, Womersley J (1974) Body fat assessed from total body density and its estimation from skinfold thickness: measurements on 481 men and women aged from 16 to 72 Years. Br J Nutr 32:77–97. 10.1079/BJN19740060 4843734 10.1079/bjn19740060

[36] Barnard N, Scialli AR, Bertron P et al (2000) Acceptability of a therapeutic low-fat, vegan diet in premenopausal women. J Nutr Educ 32:314–319. 10.1016/S0022-3182(00)70590-5

[37] Del Bo’ C, Fracassetti D, Lanti C et al (2015) Comparison of DNA damage by the comet assay in fresh versus cryopreserved peripheral blood mononuclear cells obtained following dietary intervention. Mutagenesis 30:29–35. 10.1093/mutage/geu058 25527725 10.1093/mutage/geu058

[38] Knopfholz J, Disserol CCD, Pierin AJ et al (2014) Validation of the Friedewald Formula in patients with metabolic syndrome. Cholesterol 2014:1–5. 10.1155/2014/261878 10.1155/2014/261878 PMC3941209 24672715

[39] Matthews DR, Hosker JP, Rudenski AS et al (1985) Homeostasis model assessment: insulin resistance and ?-cell function from fasting plasma glucose and insulin concentrations in man. Diabetologia 28:412–419. 10.1007/BF00280883 3899825 10.1007/BF00280883

[40] Wallace TM, Levy JC, Matthews DR (2004) Use and Abuse of HOMA Modeling. Diabetes Care 27:1487–1495. 10.2337/diacare.27.6.1487 15161807 10.2337/diacare.27.6.1487

[41] Leonard UM, Kiely ME (2024) Can micronutrient requirements be met by diets from sustainable sources: outcomes of dietary modelling studies using diet optimization. Ann Med 56(1):2389295. 10.1080/07853890.2024.2389295 39129219 10.1080/07853890.2024.2389295 PMC11321105

[42] Hughes J, Pearson E, Grafenauer S (2022) Legumes—A comprehensive exploration of global food-based dietary guidelines and consumption. Nutrients 14:3080. 10.3390/nu14153080 35956258 10.3390/nu14153080 PMC9370574

[43] Onwezen MC, Bouwman EP, Reinders MJ, Dagevos H (2021) A systematic review on consumer acceptance of alternative proteins: pulses, algae, insects, plant-based meat alternatives, and cultured meat. Appetite 159:105058. 10.1016/j.appet.2020.105058 33276014 10.1016/j.appet.2020.105058

[44] Amoah I, Ascione A, Muthanna F et al (2023) Sustainable strategies for increasing legume consumption: culinary and educational approaches. Foods 12:2265. 10.3390/foods12112265 37297509 10.3390/foods12112265 PMC10253191

[45] Lisciani S, Marconi S, Le Donne C et al (2024) Legumes and common beans in sustainable diets: nutritional quality, environmental benefits, spread and use in food preparations. Front Nutr 11:1385232. 10.3389/fnut.2024.1385232 38769988 10.3389/fnut.2024.1385232 PMC11104268

[46] Lombardo M, Ascione A, Feraco A et al (2023) Promoting legume consumption: strategies for health, nutrition, and culinary applications. Foods 26(1):65. 10.3390/Foods2023-15083

[47] Fanzo J, Davis C (2019) Can diets be healthy, sustainable, and equitable? Curr Obes Rep 8:495–503. 10.1007/s13679-019-00362-0 31654336 10.1007/s13679-019-00362-0 PMC6910888

[48] Luzzani G (2022) The sustainability of diets: current understanding and shortcomings. Curr Opin Environ Sci Health 30:100398. 10.1016/j.coesh.2022.100398

[49] Gill SK, Rossi M, Bajka B, Whelan K (2021) Dietary fibre in gastrointestinal health and disease. Nat Rev Gastroenterol Hepatol 18:101–116. 10.1038/s41575-020-00375-4 33208922 10.1038/s41575-020-00375-4

[50] Mayor S (2019) Eating more fibre linked to reduced risk of non-communicable diseases and death, review finds. BMJ l159. 10.1136/bmj.l159.

[51] Fu L, Zhang G, Qian S et al (2022) Associations between dietary fiber intake and cardiovascular risk factors: an umbrella review of meta-analyses of randomized controlled trials. Front Nutr 9:972399. 10.3389/fnut.2022.972399 36172520 10.3389/fnut.2022.972399 PMC9511151

[52] Crowe FL, Balkwill A, Cairns BJ et al (2014) Source of dietary fibre and diverticular disease incidence: a prospective study of UK women. Gut 63:1450–1456. 10.1136/gutjnl-2013-304644 24385599 10.1136/gutjnl-2013-304644 PMC4145436

[53] Stephen AM, Champ MM-J, Cloran SJ et al (2017) Dietary fibre in Europe: current state of knowledge on definitions, sources, recommendations, intakes and relationships to health. Nutr Res Rev 30:149–190. 10.1017/S095442241700004X 28676135 10.1017/S095442241700004X

[54] Sette S, Le Donne C, Piccinelli R et al (2011) The third Italian National Food Consumption Survey, INRAN-SCAI 2005–06 – Part 1: nutrient intakes in Italy. Nutr Metabolism Cardiovasc Dis 21:922–932. 10.1016/j.numecd.2010.03.001 10.1016/j.numecd.2010.03.001 20674305

[55] Wang T, Lu J, Shi L et al (2020) Association of insulin resistance and β-cell dysfunction with incident diabetes among adults in China: a nationwide, population-based, prospective cohort study. Lancet Diabetes Endocrinol 8:115–124. 10.1016/S2213-8587(19)30425-5 31879247 10.1016/S2213-8587(19)30425-5

[56] Wang Y, Liu B, Han H et al (2023) Associations between plant-based dietary patterns and risks of type 2 diabetes, cardiovascular disease, cancer, and mortality – a systematic review and meta-analysis. Nutr J 22:46. 10.1186/s12937-023-00877-2 37789346 10.1186/s12937-023-00877-2 PMC10548756

[57] Qian F, Liu G, Hu FB et al (2019) Association between plant-based dietary patterns and risk of type 2 diabetes. JAMA Intern Med 179:1335. 10.1001/jamainternmed.2019.2195 31329220 10.1001/jamainternmed.2019.2195 PMC6646993

[58] Mudryj AN, Yu N, Aukema HM (2014) Nutritional and health benefits of pulses. Appl Physiol Nutr Metab 39:1197–1204. 10.1139/apnm-2013-0557 25061763 10.1139/apnm-2013-0557

[59] Gołąbek KD, Regulska-Ilow B (2019) Dietary support in insulin resistance: an overview of current scientific reports. Adv Clin Experimental Med 28:1577–1585. 10.17219/acem/109976 10.17219/acem/109976 31756065

[60] Nolan JJ, Færch K (2012) Estimating insulin sensitivity and beta cell function: perspectives from the modern pandemics of obesity and type 2 diabetes. Diabetologia 55:2863–2867. 10.1007/s00125-012-2684-0 22911384 10.1007/s00125-012-2684-0

[61] Jenko Pražnikar Z, Šik Novak K, Bogataj Jontez N et al (2023) Inflammatory and intestinal permeability biomarkers in healthy participants on long term vegan, vegetarian, omnivore and low-carbohydrate high-fat diet. Sci Rep 13:17286. 10.1038/s41598-023-44233-0 37828090 10.1038/s41598-023-44233-0 PMC10570364

[62] Fuke N, Yamashita T, Shimizu S et al (2023) Association of plasma lipopolysaccharide-binding protein concentration with dietary factors, gut microbiota, and health status in the Japanese general adult population: a cross-sectional study. Metabolites 13:250. 10.3390/metabo13020250 36837869 10.3390/metabo13020250 PMC9965710

[63] Del Bo’ C, Bernardi S, Cherubini A et al (2021) A polyphenol-rich dietary pattern improves intestinal permeability, evaluated as serum zonulin levels, in older subjects: The MaPLE randomised controlled trial. Clin Nutr 40:3006–3018. 10.1016/j.clnu.2020.12.014 33388204 10.1016/j.clnu.2020.12.014

[64] Thomas MS, Calle M, Fernandez ML (2023) Healthy plant-based diets improve dyslipidemias, insulin resistance, and inflammation in metabolic syndrome. A narrative review. Adv Nutr 14:44–54. 10.1016/j.advnut.2022.10.002 36811593 10.1016/j.advnut.2022.10.002 PMC10103000

[65] Riso P, Martini D, Moller P et al (2010) DNA damage and repair activity after broccoli intake in young healthy smokers. Mutagenesis 25:595–602. 10.1093/mutage/geq045 20713433 10.1093/mutage/geq045

[66] Porrini M, Riso P, Brusamolino A et al (2005) Daily intake of a formulated tomato drink affects carotenoid plasma and lymphocyte concentrations and improves cellular antioxidant protection. Br J Nutr 93:93–99. 10.1079/BJN20041315 15705230 10.1079/bjn20041315

[67] Guaraldi F, Deon V, Del Bo’ C et al (2018) Effect of short-term hazelnut consumption on DNA damage and oxidized LDL in children and adolescents with primary hyperlipidemia: a randomized controlled trial. J Nutr Biochem 57:206–211. 10.1016/j.jnutbio.2018.03.012 29753234 10.1016/j.jnutbio.2018.03.012

[68] Riso P, Klimis-Zacas D, Del Bo’ C et al (2013) Effect of a wild blueberry (Vaccinium angustifolium) drink intervention on markers of oxidative stress, inflammation and endothelial function in humans with cardiovascular risk factors. Eur J Nutr 52:949–961. 10.1007/s00394-012-0402-9 22733001 10.1007/s00394-012-0402-9

[69] Lagunas-Rangel FA, Bermúdez-Cruz RM (2020) Natural compounds that target DNA repair pathways and their therapeutic potential to counteract cancer cells. Front Oncol 10:598174. 10.3389/fonc.2020.598174 33330091 10.3389/fonc.2020.598174 PMC7710985

[70] De Filippo C, Cavalieri D, Di Paola M et al (2010) Impact of diet in shaping gut microbiota revealed by a comparative study in children from Europe and rural Africa. Proc Natl Acad Sci 107:14691–14696. 10.1073/pnas.1005963107 20679230 10.1073/pnas.1005963107 PMC2930426

[71] David LA, Materna AC, Friedman J et al (2014) Host lifestyle affects human microbiota on daily timescales. Genome Biol 15:R89. 10.1186/gb-2014-15-7-r89 25146375 10.1186/gb-2014-15-7-r89 PMC4405912

[72] Clavel T, Desmarchelier C, Haller D et al (2014) Intestinal microbiota in metabolic diseases. Gut Microbes 5:544–551. 10.4161/gmic.29331 25003516 10.4161/gmic.29331

[73] Davison KM, Temple NJ (2018) Cereal fiber, fruit fiber, and type 2 diabetes: explaining the paradox. J Diabetes Complications 32:240–245. 10.1016/j.jdiacomp.2017.11.002 29191432 10.1016/j.jdiacomp.2017.11.002

[74] Pellegrini B, Strootman LX, Fryganas C, Martini D, Fogliano V (2025) Home-made vs industry-made: Nutrient composition and content of potentiallyharmful compounds of different food products. Curr Res Food Sci 10:100958. 10.1016/j.crfs.2024.100958 39811255 10.1016/j.crfs.2024.100958 PMC11730957

[75] (2012) Cadmium dietary exposure in the European population. EFSA J 10:2551. 10.2903/j.efsa.2012.2551 10.2903/j.efsa.2012.2831 PMC13092898 42016114

[76] Hejna M, Gottardo D, Baldi A, Dell’Orto V, Cheli F, Zaninelli M, Rossi L (2018) Review: Nutritional ecology of heavy metals. Animal 12(10):2156–2170. 10.1017/S175173111700355X 29306340 10.1017/S175173111700355X

[77] Zhao D, Wang P, Zhao F-J (2024) Toxic metals and metalloids in food: current status, health risks, and mitigation strategies. Curr Environ Health Rep 11:468–483. 10.1007/s40572-024-00462-7 39352604 10.1007/s40572-024-00462-7 PMC11588791

[78] European Commission (2006) Commission Regulation (EC) No 1881/2006 of 19 December 2006 setting maximum levels for certain contaminants in foodstuffs. Off J Eur Union

[79] Rai PK, Lee SS, Zhang M et al (2019) Heavy metals in food crops: health risks, fate, mechanisms, and management. Environ Int 125:365–385. 10.1016/j.envint.2019.01.067 30743144 10.1016/j.envint.2019.01.067

[80] Società Italiana Valori di Riferimento (SIVR) (2017) Fourth list of reference values for elements, organic compounds and their metabolites

[81] (2015) Scientific Opinion on the risks to public health related to the presence of nickel in food and drinking water. EFSA J 13. 10.2903/j.efsa.2015.4002

[82] Babaahmadifooladi M, Jacxsens L, Van de Wiele T et al (2021) Assessment of bioaccessible and dialyzable fractions of nickel in food products and their impact on the chronic exposure of Belgian population to nickel. Food Chem 342:128210. 10.1016/j.foodchem.2020.128210 33508898 10.1016/j.foodchem.2020.128210

[83] Beal T, Ortenzi F, Fanzo J (2023) Estimated micronutrient shortfalls of the EAT–Lancet planetary health diet. Lancet Planet Health 7:e233–e237. 10.1016/S2542-5196(23)00006-2 36889864 10.1016/S2542-5196(23)00006-2

[84] EFSA (2026) Chemical contaminants in food and feed.Available at (accessed online on 31 May 2026) https://www.efsa.europa.eu/en/topics/topic/chemical-contaminants-food-feed

[85] Italian Society for Reference Values (SIVR) (2017) Fourth List of Reference Values for Elements, Organic Compounds and Their Metabolites. Italy. https://www.sivr.it/

